# FSH/LH-Dependent Upregulation of Ahr in Murine Granulosa Cells Is Controlled by PKA Signaling and Involves Epigenetic Regulation

**DOI:** 10.3390/ijms20123068

**Published:** 2019-06-23

**Authors:** Antti Matvere, Indrek Teino, Inge Varik, Sulev Kuuse, Tarmo Tiido, Arnold Kristjuhan, Toivo Maimets

**Affiliations:** 1 Department of Cell Biology, Institute of Molecular and Cell Biology, University of Tartu, Riia 23, 51010 Tartu, Estonia; indrek.teino@ut.ee (I.T.); inge.varik@gmail.com (I.V.); skuuse@ebc.ee (S.K.); arnold.kristjuhan@ut.ee (A.K.); toivo.maimets@ut.ee (T.M.); 2 Clinical Research Centre, National Centre of Translational and Clinical Research, University of Tartu, Ravila 19, 50411 Tartu, Estonia; tarmo.tiido@ut.ee

**Keywords:** aryl hydrocarbon receptor (AhR), follicle-stimulating hormone (FSH), luteinizing hormone (LH), post-transcriptional regulation, protein kinase A (PKA), chromatin remodeling

## Abstract

The aryl hydrocarbon receptor (Ahr) is a ligand-activated transcription factor primarily known for its toxicological functions. Recent studies have established its importance in many physiological processes including female reproduction, although there is limited data about the precise mechanisms how Ahr itself is regulated during ovarian follicle maturation. This study describes the expression of Ahr in ovarian granulosa cells (GCs) of immature mice in a gonadotropin-dependent manner. We show that Ahr upregulation in vivo requires both follicle stimulating hormone (FSH) and luteinizing hormone (LH) activities. FSH alone increased *Ahr* mRNA, but had no effect on Ahr protein level, implicating a possible LH-dependent post-transcriptional regulation. Also, the increase in Ahr protein is specific to large antral follicles in induced follicle maturation. We show that *Ahr* expression in GCs of mid-phase follicular maturation is downregulated by protein kinase A (PKA) signaling and activation of *Ahr* promoter is regulated by chromatin remodeling.

## 1. Introduction

The aryl hydrocarbon receptor (Ahr) is a ligand-activated transcription factor regulating a set of genes in multiple species and tissues [1]. It contains a basic helix-loop-helix (bHLH) and Per-Arnt-Sim (PAS) domain, responsible for its DNA-binding and ligand binding, respectively [2]. Ahr can be activated by a variety of ligands, among them environmental contaminants such as polycyclic and halogenated aromatic hydrocarbons (PAH and HAHs), including the high-affinity agonist 2,3,7,8-tetrachlorodibenzo-p-dioxin (TCDD) [3]. In an inactive state, the unliganded Ahr is located in the cytoplasm forming a complex with proteins Hsp90, p23 and Xap2 [4,5]. After binding to its ligand, Ahr translocates to the nucleus [1] and dimerizes with the aryl hydrocarbon nuclear translocator (Arnt). The formed complex then binds to the Ahr response element in the promoter of target genes and activates expression. Among these genes are those responsible for detoxifying xenobiotics (*Cyp1a1*, *Cyp1b1*), but also its own repressor aryl hydrocarbon receptor repressor (Ahrr) necessary for feedback loop control [6,7,8].

Findings establishing Ahr as an evolutionarily conserved protein [9] have indicated that apart from mediating the toxicity of pollutants, Ahr may also have different roles in normal physiology. Among the essential functions of Ahr in the immune system and liver [10,11], in cell cycle regulation and tumorigenesis [12,13], this protein is also shown to be important in reproduction. Earlier studies with female rats showed TCDD to be an endocrine disruptor by blocking ovulation and causing disturbances in steroid hormone production in Ahr-dependent manner [14,15]. More recently, the role of Ahr in regulating female reproduction has become evident with studies using Ahr knockout mice. Specifically, these mice have reproductive defects such as reduced number of antral follicles [16], slower ovarian follicle growth and reduced ability to ovulate [12,17] as well as reduced number of corpora lutea [18]. AhrKO mice are shown to have difficulties in maintaining pregnancies and although fertile, they exhibit smaller litter size compared to WT mice [2,19]. AhrKO mice have decreased ability to produce estradiol in the ovary along with reduced responsiveness of antral follicles to gonadotropins—follicle stimulating hormone (FSH) and luteinizing hormone (LH)—due to reduced expression of FSH receptor (*Fshr*) and luteinizing hormone/choriogonadotropin receptor (*Lhcgr*) [17,19].

Ovarian maturation of follicles to a preovulatory stage requires precisely regulated hormonal control by FSH and LH. The action of these glycoprotein gonadotropins is mediated by corresponding FSH and LH receptors [20]. FSH receptors are expressed on granulosa cells (GCs) at all stages of follicle maturation, while the expression of LH receptors is limited to the thecal and granulosa cells of follicles that have reached the antral stage [21,22]. FSH initiates the maturation of follicles by regulating a battery of genes resulting in GC differentiation, a hallmark of which is the increased expression of *Cyp19a1* (encoding aromatase involved in estrogen production) and *Lhcgr*. While FSH together with other paracrine factors regulates the development of primary follicles to preantral and antral stages, Lhcgr signaling becomes more prevalent as a result of FSH stimulation in the second half of follicular growth phase [23]. Together with increased estradiol production and expression of Lhcgr, an LH-surge is induced, leading to ovulation of the oocyte [24,25].

Both Fshr and Lhcgr are transmembrane G-protein-coupled receptors, which upon receptor occupancy stimulate adenylyl cyclase resulting in increased intracellular cAMP levels and activation of protein kinase A (PKA) [26,27]. Although different intracellular signaling cascades are shown to be activated by this mechanism, most of the actions of FSH on GC differentiation and follicle maturation are believed to be mediated by PKA [28].

The importance of FSH, LH and estradiol in folliculogenesis is the reason why even the slightest aberrances from their highly controlled balance may cause reproductive defects [29]. Considering the role of Ahr in influencing hormonal signaling, it is of utmost importance to understand if Ahr itself is regulated by gonadotropins during follicular maturation. Chaffin et al. showed that in macaque granulosa cells *Ahr* was induced in response to LH in the periovulatory phase of the follicular cycle [30]. Contrarily, in mature rat granulosa cells, *Ahr* was shown to be upregulated during follicle maturation, but downregulated following the LH surge [31]. In accordance with this, we have previously shown downregulation of *Ahr* expression in response to an ovulatory dose of LH in mice [32].

As of today, some progress has been made in specifying the functional role of Ahr in the ovary. However, there are very little data on the molecular mechanisms behind *Ahr* expression. We have previously shown that downregulation of Ahr in GCs of preovulatory follicles after the LH surge is dependent on PKA signaling [32]. Moreover, *Ahr* expression decreased due to reduced transcription rate but not due to changes in mRNA stability. Finally, we provided evidence that the regulation of *Ahr* involves epigenetic mechanisms. More precisely, *Ahr* was repressed by chromatin condensation at the promoter. Our data befittingly complies with the increased knowledge of epigenetic control of gene regulation in the ovary [33]. Furthermore, regulation of Ahr by chromatin dependent mechanisms has also been reported by several studies [34,35].

New data describing the role of Ahr in normophysiology and disease are accumulating to date. Mostly these studies are concentrating on the modulation of Ahr activity by various agonists/antagonists. However, the mechanisms regulating the expression of Ahr are less understood and need closer scrutiny. In our previous study, we investigated Ahr expression in preovulatory GCs after the LH surge. Our aim in this study was to characterize the expression profile of Ahr in GCs during follicular maturation before the LH surge and elucidate the underlying mechanisms. We show that Ahr upregulation requires both gonadotropin-FSH and LH-activities and that Ahr at protein level is mainly upregulated in large antral follicles. We demonstrate that *Ahr* expression in GCs is controlled by PKA signaling pathway and activation of *Ahr* promoter includes epigenetic regulation.

## 2. Results

### 2.1. The Upregulation of Ahr in GCs during Follicular Maturation Requires both FSH and LH Activity

**Figure 1:**
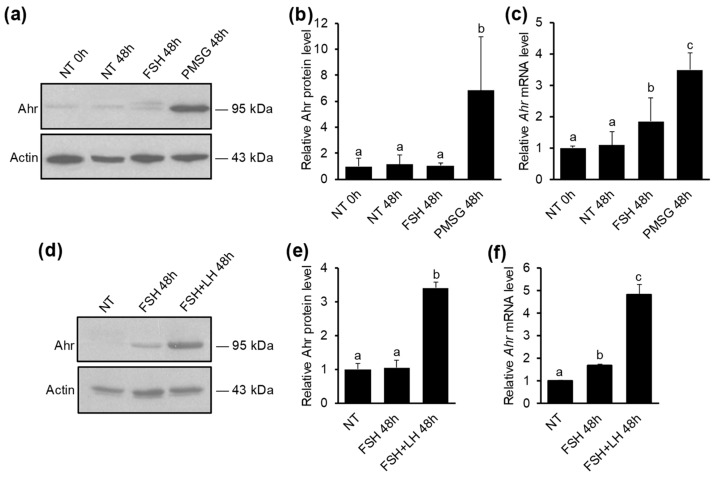
The effect of gonadotropins on Ahr expression in ovarian GCs in vivo. (**a**) Mice were injected once with 5 IU of PMSG, 5 IU of FSH or vehicle (NT) and lysates were collected from granulosa cells (GCs) isolated before (0 h) or 48 h later. Representative Western blot shows Ahr and actin protein levels. Specificity of Ahr antibody is shown in Supplementary Figure S1d). (**b**) Densitometry analysis of three independent experiments (mean ± SD) representing Ahr protein levels normalized to actin. (**c**) qPCR analysis of *Ahr* mRNA levels. (**d**) Mice were injected in total 4 times (every 12 h) with FSH (1.5 IU) or FSH (1.5 IU) + LH (1.25 IU) and lysates were collected from GCs isolated before (NT) or 48 h after initial injection. Representative Western blot shows Ahr and actin protein levels. (**e**) Densitometry analysis of three independent experiments (mean ± SD) representing Ahr protein levels normalized to actin. (**f**) qPCR analysis of *Ahr* mRNA levels. Bars with no common superscripts are significantly different (*p* < 0.05).

Pregnant mare’s serum gonadotropin (PMSG) is a gonadotropin known to have intrinsic FSH but also residual LH activity and is commonly used for inducing superovulation and maturation of GCs in the ovary. To investigate if PMSG influences Ahr expression and whether it is caused solely by its FSH activity, mice were injected with 5 IU PMSG or 5 IU FSH. 48 h later ovaries were excised and GCs extracted. Western blot analysis showed that PMSG, but not FSH, elevates Ahr protein levels 6.8-fold compared to vehicle-treated (NT) mice (Figure 1a,b). Similar results were obtained by *Ahr* mRNA analysis, which showed a 3.5-fold upregulation of *Ahr* expression in PMSG-treated animals (Figure 1c). Interestingly, a statistically significant upregulation of *Ahr* mRNA was also caused by FSH, although to a smaller extent (1.9-fold). Injecting immature mice with 5 IU LH or 5 IU hCG (human chorionic gonadotropin—LH analog) had no effect on *Ahr* expression in GCs (Supplementary Figure S1a). To clarify, whether the discrepancy between the effect of FSH on Ahr protein and mRNA levels is caused by short half-life of FSH, but also to study, if upregulation of Ahr protein requires the activity of both gonadotropins, we injected mice in total four times (every 12 h) with FSH (1.5 IU) alone or simultaneously with LH (1.25 IU). 48 h after the first injection, GCs were extracted and subjected to further analysis. Western blot analysis showed that FSH treatment alone did not influence Ahr protein levels (Figure 1d,e). However, Ahr protein level was elevated 3.5-fold when mice received FSH and LH in combination. Analysis of *Ahr* mRNA confirmed that LH activity is indeed important resulting in 4.8-fold upregulation of *Ahr* in response to combined hormone treatment (Figure 1f). Again, FSH alone induced modest (1.7-fold), but statistically significant increase in *Ahr* expression. We also measured *Ahr* hnRNA levels, which due to its short half-life is considered a suitable indicator of transcription rate, and noted a similar pattern to changes in mRNA (Supplementary Figure S1b). Additionally, the upregulation of maturation marker genes was detected in response to combined hormonal treatment (Supplementary Figure S1c). Taken together, we found that *Ahr* is upregulated in GCs during follicular maturation and the upregulation requires both FSH and LH activity. Since PMSG treatment provided similar results with combined treatment of FSH and LH, we used PMSG in our further experiments to study Ahr expression during follicle maturation.

### 2.2. The Effect of PMSG on The Expression Dynamics of Ahr and Follicle Maturation Marker Genes

**Figure 2:**
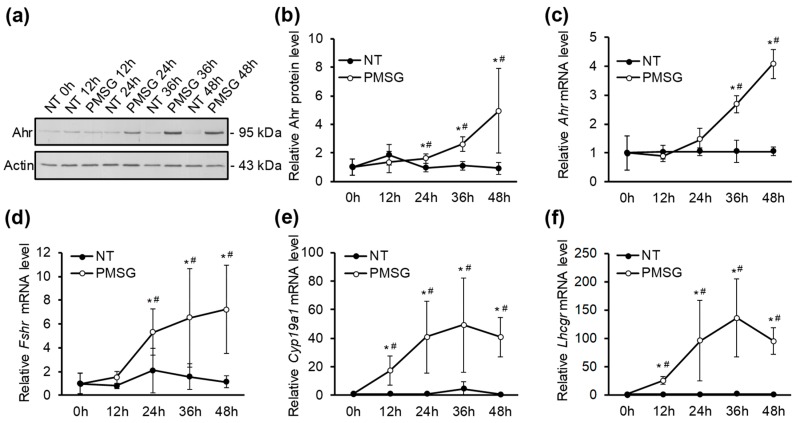
The effect of PMSG on expression dynamics of Ahr and follicle maturation marker genes in GCs in vivo. (**a**) Mice were injected with 5 IU of PMSG or vehicle (NT) and lysates were collected from GCs isolated before (0 h) or up to 48 h later. Representative Western blot shows Ahr and actin protein levels. (**b**) Densitometry analysis of five independent experiments (mean ± SD) representing Ahr protein levels normalized to actin. qPCR analysis of *Ahr* (**c**), *Fshr* (**d**), *Cyp19a1* (**e**) and *Lhcgr* (**f**) mRNA in GCs isolated before (0 h) or up to 48 h after PMSG (5 IU) or vehicle (NT) treatment. * *p* < 0.05 vs. NT; # *p* < 0.05 vs. 0 h.

In order to further clarify the mechanisms of how Ahr is regulated in GCs during follicular maturation, we investigated the temporal pattern of Ahr expression. For this, mice were injected with PMSG or vehicle (NT) and GCs were extracted every 12 h up to 48 h post-injection. Western blot analysis showed that Ahr protein levels are significantly higher from its control 24 h after PMSG injection (1.6-fold) and gradually increase up to 48 h (4.9-fold) (Figure 2a,b). Analysis of mRNA expression showed a similar pattern with 4.1-fold upregulation after 48 h (Figure 2c).

It is well established that follicle maturation and GC differentiation is accompanied by increased expression of several marker genes, most notably *Fshr*, *Cyp19a1* and *Lhcgr* [36,37,38,39,40,41,42]. In order to validate hormonal stimulation, we measured the expression level of these genes in ovarian GCs extracted every 12 h up to 48 h after PMSG treatment. The results demonstrate that PMSG stimulation leads to a time-dependent upregulation of all three marker genes compared to NT controls (Figure 2d–f). A significant 5.3-fold increase in *Fshr* transcript levels occurred after 24 h and continued to increase up to 7.2-fold 48 h after PMSG injection (Figure 2d). *Cyp19a1* mRNA levels were elevated almost 20-fold after 12 h and resulted in about 40-fold increase 48 h after hormone treatment (Figure 2e). PMSG increased *Lhcgr* expression approximately 25-fold after 12 h and resulted in almost 95-fold increase 48 h after treatment (Figure 2f). Maximal *Cyp19a1* and *Lhcgr* mRNA levels were measured 36 h post-injection (approximately 50- and 130-fold, respectively), followed by a slight trend of decline up to 48 h post-PMSG (Figure 2e,f). There were no significant mRNA level changes in vehicle-treated mice.

Previous studies have shown that the role of Ahr in the ovary may be attributed, at least partly, to regulating the expression of *Cyp19a1* and thereby contributing to estradiol production [12,19]. There is also evidence that Ahr directly regulates the expression of *Fshr* by binding to its promoter [17,43]. According to these data, we aimed to characterize the temporal relationship between the expression of Ahr and abovementioned genes. We found that the upregulation of Ahr occurred comparatively late compared to *Fshr*, *Cyp19a1* and *Lhcgr*.

### 2.3. Ahr Is Upregulated in Large Antral Follicles in Response to PMSG

**Figure 3:**
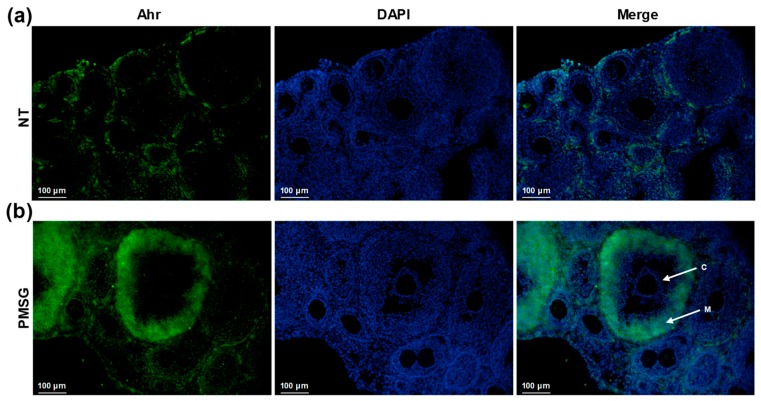
The effect of PMSG treatment on Ahr localization in ovary in vivo. Mice were injected with 5 IU of PMSG or vehicle (NT) and ovaries were isolated 48 h later. Representative images of immunofluorescence analysis of vehicle-treated (**a**) and PMSG-treated (**b**) ovaries from three independent experiments using Ahr-specific antibody (green). Nuclei were stained with DAPI (blue). Normal rabbit IgG as primary antibody was used for isotype control (Supplementary Figure S2). C—cumulus granulosa cells. M—mural granulosa cells. Scale bar, 100 μm.

To investigate the spatial distribution of Ahr in the ovary, mice were treated with 5 IU PMSG, ovaries excised and cryosections subjected to immunofluorescence analysis. The results show that Ahr is detectable to a small extent in NT samples (Figure 3a), as seen previously by Western blot analysis (Figure 1a and Figure 2a). PMSG treatment results in follicular maturation, illustrated by the presence of large antral follicles. Moreover, Ahr is highly upregulated in antral follicles, particularly in mural GCs (Figure 3b). Cumulus GCs, however, seem to lack Ahr. Collectively, these results demonstrate that PMSG does induce follicular maturation in our experiments, as shown by augmented expression of maturation marker genes (Figure 2d–f) and increased number of antral follicles in the ovary (Figure 3b).

### 2.4. Ahr Is Downregulated by Protein Kinase A Signaling Pathway

**Figure 4:**
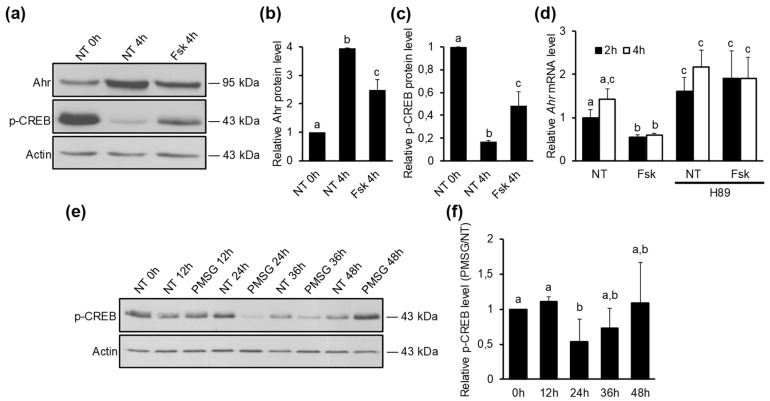
The effect of PKA signaling on *Ahr* expression. (**a**) Mice were treated with 5 IU of PMSG for 24 h in vivo, GCs were isolated and cultured in vitro. Following attachment (2–3 h later), GCs were treated with vehicle (NT) or Fsk (10 μM). Representative Western blot shows Ahr, p-CREB and actin levels in GCs before (0 h) and 4 h after vehicle (NT) or forskolin (Fsk, 10 μM) treatment. (**b**) Densitometry analysis of three independent experiments (mean ± SD) representing Ahr protein levels normalized to actin. (**c**) Densitometry analysis of three independent experiments (mean ± SD) representing p-CREB protein levels normalized to actin. (**d**) Mice were treated with 5 IU of PMSG for 24 h in vivo, GCs were isolated and cultured in vitro. Following attachment (2–3 h later), GCs were treated with vehicle (NT) or Fsk (10 μM) alone or in combination with PKA inhibitor H89 (10 μM). *Ahr* mRNA levels were measured 2 h and 4 h later by qPCR. (**e**) Representative Western blot shows p-CREB and actin protein levels measured in GCs isolated before (0 h) or up to 48 h after PMSG (5 IU) or vehicle treatment in vivo. (**f**) Densitometry analysis of p-CREB protein levels. The data are presented as ratio of PMSG vs, NT for different timepoints from three independent experiments (mean ± SD). Bars with no common superscripts are significantly different (*p* < 0.05).

Maturation of ovarian follicles to a preovulatory stage requires FSH signaling [44]. It is also well-established that most of the actions of FSH are mediated by cAMP formation and activation of protein kinase A (PKA) [28,45]. To investigate whether PKA signaling pathway is regulating Ahr expression, follicle maturation was induced by 5 IU PMSG and ovaries were excised 24 h later, which is the time when Ahr levels in the ovary start to increase (Figure 2b). PKA activity in GCs was monitored by measuring the levels of phosphorylated cAMP-response element-binding protein (p-CREB), which is a well-known direct protein target of PKA [26,46]. After 4h of GC culture, we saw a substantial decrease (83%) in p-CREB levels (Figure 4a,c) along with increased Ahr protein (3.9-fold) compared to 0 h control (Figure 4a,b), suggesting that a decrease in PKA activity may be necessary to elevate Ahr. Forskolin is a compound known to activate adenylyl cyclase and increase cellular cAMP levels, resulting in subsequent activation of PKA [47,48]. When GCs were treated 4h with Fsk, we observed a significant decrease (38%) in Ahr (Figure 4a,b) and a 2.8-fold increase in p-CREB (Figure 4a,c) protein levels compared to the respective controls, hence supporting the role of PKA signaling in repression of Ahr.

The observed decline in p-CREB levels and thus PKA activity after GC culture may be caused by the loss of gonadotropin stimulus, which was present in vivo. To verify this, PKA activity was additionally evaluated by measuring the mRNA levels of *Fshr*, *Cyp19a1* and *Lhcgr*, since the expression of these genes is shown to be dependent on PKA signaling [48,49]. Our data show a rapid downregulation of all three genes after the start of granulosa cell culture, indicative of diminished PKA activity in vitro. Conversely, Fsk treatment increased the expression of these genes compared to NT controls at 12 h and 24 h timepoints (Supplementary Figure S3).

The effect of forskolin on Ahr downregulation was also confirmed by mRNA analysis, which showed a significant decrease (44%) in *Ahr* expression already 2 h after forskolin treatment followed by a 58% decrease at 4 h (Figure 4d). In order to corroborate that the regulation of *Ahr* expression was directly due to PKA activation, GCs were treated with PKA inhibitor H89 in combination with vehicle (NT) or Fsk. We found that H89 abolished the repressive effect of Fsk on *Ahr* mRNA (Figure 4d), confirming that downregulation of *Ahr* transcription requires PKA signaling. After further culture of granulosa cells, we detected a gradual increase in *Ahr* mRNA (Supplementary Figure S4a) and hnRNA (Supplementary Figure S4b) levels in non-treated cells up to 24 h. The effect of Fsk on *Ahr* repression was also evident up to this time (Supplementary Figure S4a,b).

To assess PKA activity in GCs during follicular maturation in vivo, mice were injected with 5 IU PMSG or vehicle (NT) and protein lysates collected at various timepoints before (0 h) or up to 48 h after the injection, followed by Western blot analysis of p-CREB. The presence of p-CREB was detected in GCs of non-treated mice, indicating basal PKA activity in ovarian GCs in vivo (Figure 4e). However, as shown by densitometry analysis, PMSG stimulation resulted in a significant decrease of p-CREB levels 24 h post-injection, at the time when Ahr expression starts to elevate (Figure 4f). At 36 h and 48 h timepoints this effect was abolished.

Collectively, the results of our experiments show that PKA signaling reduces *Ahr* expression in mid-phase follicular GCs in vitro. Additionally, this circumstance appears to be present in vivo, where PKA signaling is weakened as Ahr levels are starting to increase.

### 2.5. The Increased Transcription of Ahr in Response to PMSG is Regulated by Chromatin Accessibility

**Figure 5:**
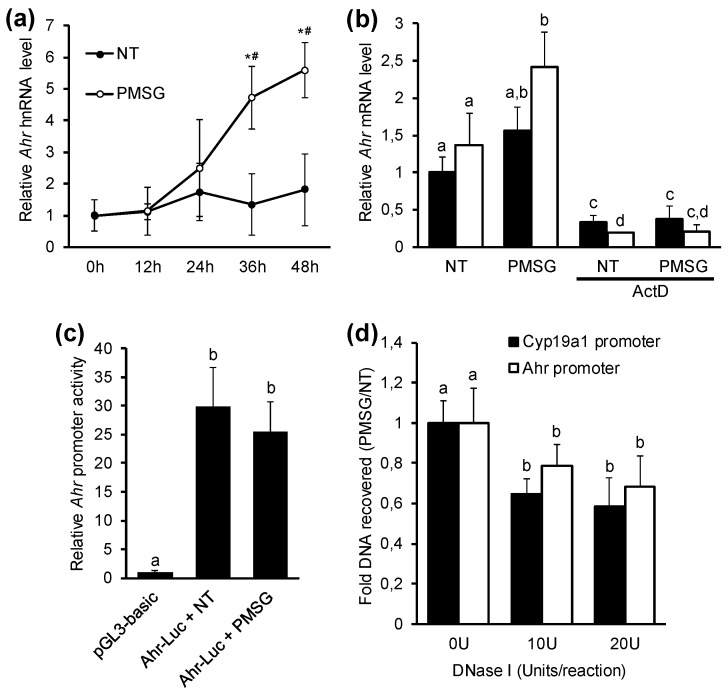
The effect of pregnant mare’s serum gonadotropin (PMSG) on *Ahr* gene expression mechanisms. (**a**) *Ahr* heteronuclear RNA (hnRNA) levels were measured by qPCR in GCs isolated before (0 h) or up to 48 h after PMSG (5 IU) or vehicle (NT) treatment in vivo. Data are presented as means ± SD from five independent experiments. * *p* < 0.05 vs NT; # *p* < 0.05 vs. 0 h. (**b**) Mice were injected with 5 IU of PMSG, GCs were isolated 48 h later and cultured in vitro. Following attachment (2–3 h later), cells were incubated with vehicle (NT) or PMSG (5 IU/mL) alone or in combination with transcription inhibitor actinomycin D (ActD, 1 μg/mL). *Ahr* mRNA levels were measured 2 h and 4 h later by qPCR. Data are presented as means ± SD from three independent experiments. (**c**) GCs isolated from immature mice were cultured in vitro, transfected with empty vector (pGL3-basic) or *Ahr*-luciferase reporter constructs and treated with vehicle (NT) or PMSG (5 IU/mL) for 48 h followed by analysis of luciferase activity. Data are presented as means ± SD from three independent experiments. (**d**) Mice were injected with 5 IU of PMSG or vehicle (NT) and GCs were isolated 48 h later. DNA from nuclei treated with increasing concentrations of DNase I was analyzed by qPCR. The recovered DNA at promoter sites of interest were normalized to recovered genomic DNA from *Pax7* promoter, the data are presented as ratio of PMSG vs. NT from five independent experiments (mean ± SD). Bars with no common superscripts are significantly different (*p* < 0.05).

Gene expression at mRNA level can be regulated by two fundamental cellular processes—transcription and mRNA degradation. It was in our interest to elucidate whether the PMSG-induced increase in *Ahr* transcript levels occurs by increased transcription rate or by decreased mRNA degradation. To study the effect of PMSG on the rate of mRNA synthesis, we first measured the hnRNA levels in GCs of PMSG and vehicle-treated mice. GCs were obtained at various timepoints and hnRNA levels were measured by qPCR using primers designed on exon-intron border as previously described [32]. Results show that PMSG induced a significant 4.7-fold upregulation of *Ahr* hnRNA after 36 h, followed by 5.6-fold increase 48 h post-PMSG (Figure 5a). To assess the potential influence of PMSG on *Ahr* mRNA stability, we cultured GCs from PMSG-primed (48 h) mice in vitro and studied the effect of PMSG on *Ahr* mRNA levels alone or in combination with actinomycin D (ActD), which is known to abolish transcription in cells. GCs were collected at this point in time because of the abundance of Ahr protein, mRNA, but also hnRNA, indicative of active transcription. We observed a significant increase in the amount of *Ahr* transcripts after 4 h if PMSG was added to medium (Figure 5b). This increase was abrogated in the presence of ActD. Moreover, there was no difference in ActD + NT vs. ActD + PMSG treatments, suggesting that PMSG does not augment *Ahr* mRNA stability, thus supporting the evidence that PMSG-induced *Ahr* expression in ovarian GCs is regulated via increased mRNA synthesis.

*Ahr* promoter is known to contain several regulatory elements that can be targeted by downstream modulators of gonadotropin activity [50,51]. To study if PMSG induces *Ahr* expression via increased promoter activity, we transfected GCs from non-treated immature mice with a luciferase reporter construct containing *Ahr* promoter site. The 1792 bp *Ahr* promoter sequence (−1425 to +367 bp, relative to the TSS) was constructed as previously described [32]. Several studies have shown that this region contains necessary elements for constitutive *Ahr* promoter activity [50,51]. Cells were treated with 5 IU/mL PMSG. Relative luciferase activity was measured 48 h later. Our data show that PMSG has no significant effect on *Ahr* promoter activity when compared to non-treated control (Figure 5c), signifying the involvement of other regulatory mechanisms.

Several studies have demonstrated the epigenetic control of *Ahr* gene expression [34,52,53]. Recently, we showed that murine *Ahr* in response to hCG (LH analog) in preovulatory follicles is downregulated by chromatin remodeling [32]. Considering the bivalent characteristics of *Ahr* promoter, as illustrated by Ko et al. [35], we aimed to investigate whether upregulation of Ahr during follicle maturation may be controlled by changes in chromatin structure at *Ahr* promoter, allowing accessibility for transcriptional machinery required for gene expression. For this, immature mice were injected with 5 IU PMSG or vehicle and GCs isolated 48 h later. For evaluating dose-dependent effects, two concentrations of DNase I (10U and 20U) were used relative to 0U control. The state of chromatin structure was assessed by subsequent analysis of DNase I accessibility to the region −176 to −77 bp of *Ahr* promoter. This region has been shown to be influenced during modulation of *Ahr* expression previously [32]. Accessibility of this region to transcription machinery is essential for gene expression and thus makes it a good surrogate to evaluate chromatin condensation. The data are presented as the fold change of DNA recovered from GCs of PMSG vs. NT animals. The results show a significant decrease in the amount of DNA recovered from GCs of PMSG-treated mice, indicating open chromatin at *Ahr* promoter (Figure 5d). Due to the evidence that *Cyp19a1* is downregulated in preovulatory GCs in response to hCG by chromatin condensation at the promoter [54] and due to the increase in *Cyp19a1* expression in response to PMSG (Figure 2e), we used it as a positive control. We demonstrate the increased accessibility of DNase I to the promotor region of *Cyp19a1*, indicated by reduced amount of recovered DNA from GCs of PMSG-primed mice (Figure 5d). We did not see any effect of PMSG on DNase I accessibility to the promoter region of *Pax7* when compared to NT control (Supplementary Figure S5). Taken together, we show that PMSG increases *Ahr* transcription in maturing GCs by chromatin remodeling at *Ahr* promoter site.

## 3. Discussion

Recent studies have established the endogenous role of Ahr, including its significance in female reproduction. Studies with Ahr knockout mice have provided substantial evidence of its importance in regulating various physiological processes in the ovary. Considering the important biological role of Ahr in folliculogenesis, it is essential to also understand the mechanisms by which Ahr itself is regulated. Unfortunately, the data on the latter during follicle maturation is still largely unknown. The present work provides several original findings on this matter.

There are some studies that have investigated Ahr expression in periovulatory GCs after LH stimulus [30,31], but there is limited data on Ahr expression during follicle maturation, a process mainly driven by FSH. Bussman and Baranao showed that Ahr in GCs is decreased by FSH, but their experiments were performed in vitro after treating rats with estrogen analog diethylstilbestrol (DES) [55]. DES stimulates smaller follicles to grow to antral stage and this effect is not present in vivo in immature animals.

Previous work by Chaffin et al. demonstrated that *Ahr* mRNA levels increase during the follicle maturation period in normally cycling rats [31]. Additionally, it has been shown that PMSG, a hormone commonly used for inducing superovulation, increases *Ahr* expression [19,32]. PMSG has FSH activity with residual LH activity and these hormones are the main gonadotropins that coordinate follicular maturation [56]. For this reason, our aim was to find out, whether FSH activity, the main stimulator of follicle growth and maturation, is responsible for Ahr induction, or is LH activity also needed. To study the effect of gonadotropins on *Ahr* expression we used immature mice who lack naturally occurring reproductive cycle. Our results show that both FSH and LH signaling are required to elevate Ahr protein levels, since injecting mice with FSH and LH simultaneously or with PMSG, but not FSH or LH separately, resulted in a significant increase in Ahr protein levels in preovulatory granulosa cells (Figure 1). The combined treatment also resulted in elevated mRNA levels similar to the extent reported by Chaffin et al. [31]. FSH alone increased *Ahr* mRNA levels, but had no effect on the amount of Ahr protein (Figure 1). Our data emphasize the importance of LH signaling in elevation of Ahr protein levels. LH has an important role in follicle maturation distinct from the role of FSH [57]. The expression of LH receptors during follicle maturation starts to occur in GCs of antral follicles [23], present in later stages of follicular maturation. This distinct role is also endorsed by its effect on *Ahr* expression in our experiments. On the other hand, it is clear that LH (or hCG) alone without FSH, which initiates GC differentiation, is not sufficient to induce *Ahr* expression (Supplementary Figure S1a).

FSH had no effect on Ahr protein levels, but clearly increased *Ahr* transcription (Figure 1c). We first hypothesized that this is due to the short half-life of FSH, but injecting mice with FSH 4 times (every 12 h) resulted in a similar outcome as both *Ahr* mRNA (Figure 1f) as well as hnRNA (Supplementary Figure S1b) expression increased. One possibility why FSH is unable to elevate Ahr protein might be that FSH action alone is not sufficient for proper follicle maturation. Indeed, it has been demonstrated that FSH treatment alone results in a smaller number of large follicles compared to FSH + LH or PMSG [58]. This notion is further corroborated by our experimental data showing that FSH treatment has a minor effect on the expression of follicle maturation marker genes (Supplementary Figure S1c). It is possible that the smaller number of antral follicles is caused by insufficient LH signaling (which coincides with *Lhcgr* expression). Recent studies suggest that LH may control the expression of a large set of genes by regulating their protein levels post-transcriptionally [59,60,61]. In preovulatory GCs a cohort of genes is regulated very rapidly in response to an ovulatory dose of LH [62]. Fiedler et al. suggested that to enable this fast-acting effect, the expression of these genes is held in a steady state by miRNAs [63]. Several studies have also demonstrated that Ahr can be controlled by miRNAs [64,65,66]. Thus, it is possible that LH may exert its positive effect on Ahr by downregulating a specific miRNA that regulates *Ahr* mRNA. However, the exact mechanisms remain to be elucidated.

Previous studies investigating Ahr in the maturing ovary lack data on how Ahr is regulated in time and space [67]. By evaluating Ahr expression in a timely manner, we show that *Ahr* mRNA and protein levels start to increase in mid-follicular phase during maturation (Figure 2a–c). This data is concordant with above-suggested notion that the increase of Ahr protein requires LH signaling.

GC differentiation and ovarian maturation was validated by measured upregulation of *Fshr, Cyp19a1*, and *Lhcgr* (Figure 2d–f). It is known that Ahr may regulate the expression of *Fshr* and *Cyp19a1* [19,43,68]. In our experimental setting, however, we see no prior increase in Ahr expression compared to *Fshr* and *Cyp19a1*. It is possible that the relatively high dose of PMSG is sufficient to upregulate these two genes by acting through different mechanisms and thus offsetting the effects Ahr may have on their expression. Nonetheless, although Ahr may not be relevant in the PMSG-induced expression of *Fshr* and *Cyp19a1*, it cannot be ruled out that Ahr might still regulate the expression of these two genes at basal level, since GCs of immature follicles still express low levels of *Fshr* and *Cyp19a1*.

There is limited data on the spatial expression of Ahr in murine ovaries. This is the first study to provide evidence that Ahr at protein level is upregulated in large antral follicles of mice (Figure 3). High expression of Ahr was seen in mural GCs, and not cumulus GCs, a finding concordant with that of Wigglesworth et al. who measured higher *Ahr* mRNA levels in mural GCs of immature mice compared to cumulus cells [69]. They also showed higher *Lhcgr* levels in the same cells, corroborating our previous suggestion on the importance of *Lhcgr* signaling on Ahr expression.

It is well known that the action of gonadotropins in GCs is facilitated by activation of adenylyl-cyclase, resulting in activation of protein kinase A. Although a cross-talk between and among different signaling cascades is required for target gene activation, PKA pathway appears to be accountable for most of the effects of FSH-Fshr signaling on GC proliferation and differentiation [28,48]. Our aim was to elucidate whether *Ahr* expression is likewise influenced by PKA. We collected granulosa cells 24 h after injecting mice with PMSG, as this is the time Ahr levels start to increase. We hypothesize that the upregulation of Ahr after GC culture in non-treated cells was caused by decreased PKA activity, as gonadotropin signaling is lost in vitro (Figure 4a,b). This suggestion is supported by a measured decline in p-CREB levels (Figure 4a,c), a well-known direct PKA target, but also by experiments showing a decrease in transcript levels of PKA-dependent genes *Fshr*, *Cyp19a1*, and *Lhcgr* (Supplementary Figure S3). Treating GCs with PKA activator forskolin decreased Ahr mRNA and protein levels. This effect was abolished by H89, a PKA inhibitor, showing that Ahr expression is indeed repressed by PKA signaling (Figure 4d). To clarify whether Ahr expression is controlled by PKA also in vivo, we evaluated the activity of this kinase pathway by measuring p-CREB levels in GCs of NT and PMSG-treated mice during follicle maturation. A significant decrease in phosphorylated CREB levels was seen at the point during maturation where Ahr expression starts to increase (Figure 4e,f). Thus, the increase in Ahr expression is likely caused by the decrease in PKA activity. Whereas total CREB protein levels have been reported to be relatively unchanged after PMSG stimulus [26,70], our findings on evaluating PKA activity by CREB’s active, phosphorylated form are in accordance with those of Maizels et al. who similarly noted a significant decrease in p-CREB levels 24 h post-PMSG treatment in rat granulosa cells in vivo [70]. Our results also show the presence of p-CREB in GCs of non-treated mice, suggesting that basal PKA activity keeps *Ahr* expression constantly at low levels.

Gonadotropins act largely via protein kinase A. PKA in turn has been shown to downregulate Ahr, making our results appear contradictory. However, it has been shown that low-dose and high-dose PKA activity may have opposite effects on cellular processes including gene regulation [40,46]. Furthermore, gonadotropins have been shown to induce opposite effects highly dependent on the stage of follicular maturation. Particularly, folliculogenesis marker genes *Cyp19a1* and *Lhcgr* are upregulated during follicular maturation, but then rapidly downregulated in response to an ovulatory dose of LH in preovulatory follicles [36,39,40]. In fact, we have previously shown that Ahr expression is rapidly decreased in preovulatory GCs in response to an ovulatory dose of hCG (LH analog) and this downregulation was dependent on PKA activation [32]. In turn, this study demonstrates the importance of LH action on upregulation of Ahr protein during follicular maturation. Since genes are often regulated by intricate network of signaling pathways, seemingly contradictory results can be explained by the possible interplay between different downstream signaling pathways involved in gene regulation. Although gonadotropins via PKA initiate and carry follicular maturation process, signaling pathways downstream of PKA may take over the positive effects on Ahr expression. Thus, decrease in PKA activity measured 24 h after PMSG seems to be the activation barrier needed for the induction of Ahr. Further upregulation may be supported by different pathways, as PKA activity is restored 48 h after hormone injection (Figure 4d).

We have shown that Ahr is upregulated during follicle maturation, but to understand the functions of this important protein in reproduction, it is also crucial to understand the mechanisms by which Ahr is regulated. We first intended to elucidate how PMSG increases *Ahr* transcription. Since cellular mRNA levels are a sum of both transcriptional activity and mRNA degradation, we measured *Ahr* heteronuclear (hnRNA) levels, which due to its short half-life is considered to be a good surrogate marker for determining transcriptional activity of a gene [71]. Our data provide evidence that *Ahr* upregulation is mostly mediated by increased transcriptional activity, as shown by increasing hnRNA amounts in response to PMSG (Figure 5a), but also by experiments showing that PMSG does not influence *Ahr* mRNA stability (Figure 5b). The relatively late increase in *Ahr* hnRNA levels (24 h post-PMSG) is also concordant with similar patterns of Ahr protein and mRNA (Figure 2a–c) dynamics.

It is known that transcriptional activation of genes by FSH involves multiple *trans*-acting factors and *cis*-acting DNA elements [39,50]. Characterization of *Ahr* promoter has revealed that it contains several regulatory elements involved in FSH-mediated control of gene expression [51]. We intended to investigate whether PMSG has an effect on *Ahr* promoter activity by measuring luciferase activity in GCs transfected with *Ahr*-promoter reporter constructs. Although it is now widely accepted that regulatory sequences necessary for promoter activity and gene expression can be located far from TSS, the promoter sequence used in this study has been shown to contain essential elements for constitutive murine *Ahr* promoter activity [50,51]. Although promoter activity was detected in non-treated cells, we did not see an additional increase in response to PMSG in these transiently transfected cells (Figure 5c). It is possible that regulatory elements may be outside of this sequence, but these results may also suggest that PMSG increases *Ahr* expression via other mechanisms. Recently, there is emerging knowledge about epigenetic control of ovarian gene expression [33]. Chromatin structure is determined by characteristic histone modifications on nucleosomes, for example acetylation or deacetylation, representing open and closed chromatin state, respectively [72]. Studies with histone deacetylation inhibitors showing changes in *Ahr* expression have provided some insight into regulation of *Ahr* promoter activity by chromatin remodeling, affecting transcription factor accessibility to regulatory *cis*-elements [34]. In the current study, we present evidence that the increase in *Ahr* expression in response to PMSG in GCs of maturing follicles is due to decreased chromatin condensation at *Ahr* promoter (Figure 5d). Our data on the lack of PMSG effect on *Ahr* promoter activity, as shown previously (Figure 5c), could thus be explained by the importance of chromatin context, which may not be functionally present in reporter constructs. Although the state of chromatin was evaluated by DNase I accessibility to a short region of *Ahr* promoter, this sequence is in the proximity of TSS, where accessibility of transcription factors is often regulated by chromatin state. Additionally, we showed previously that *Ahr* in preovulatory GCs in response to an ovulatory dose of hCG is repressed by chromatin condensation at *Ahr* promoter, whereas we detected a change in DNase I accessibility to this short region in *Ahr* promoter, but not to *Ahr* intron [32]. Nevertheless, epigenetic modifications associated with altered chromatin structure should be further studied in the future to elaborate on the exact mechanisms in *Ahr* regulation (e.g., chromatin immunoprecipitation studies combined with nucleosome-scanning assays). Some studies have implied that FSH-mediated control of chromatin structure is a result of direct phosphorylation of histones by PKA [45,73], although this modification was shown to decrease chromatin condensation. On the other hand, there are studies reporting that PKA activity is needed to keep chromatin in a condensed state [74]. Although FSH and LH exert their effect largely via PKA pathway, it is proposed that low and high levels of this kinase activity have differential effects on target gene expression in granulosa cells [40,46]. It is reasonable to suggest that Ahr upregulation in mid-phase ovarian follicle maturation occurs due to decreased effects of PKA on chromatin condensation at *Ahr* promoter. Still, more data is needed to clarify the exact mechanisms involved in gonadotropin control of Ahr via epigenetic mechanisms.

In conclusion, this study has provided significant insights on how Ahr is regulated in ovarian granulosa cells during follicle maturation in vivo. We have shown that FSH and LH are both necessary for Ahr upregulation in murine GCs and the increase in Ahr protein is mainly restricted to large antral follicles. Additionally, we showed that *Ahr* expression is repressed by protein kinase A pathway and activation of *Ahr* promoter is controlled by chromatin remodeling.

## 4. Materials and Methods

### 4.1. Medium, Chemicals, and Antibodies

Medium DMEM/Hams F-12 was purchased from Corning (Corning, NY, USA). Pregnant mare serum gonadotropin (PMSG) and human chorionic gonadotropin (hCG) was obtained from Intervet (Boxmeer, Netherlands), follicle stimulating hormone (FSH, Follitropin alfa) and luteinizing hormone (LH, Lutropin alfa) from Merck Serono GmbH (Darmstadt, Germany), human fibronectin from BD Biosciences (Bedford, MA, USA), Forskolin (Fsk) from Tocris (Minneapolis, MN, USA), actinomycin D (ActD) from Applichem (Darmstadt, Germany) and H89 from Santa Cruz Biotechnology (Santa Cruz, CA, USA). The polyclonal Ahr antibody (SA-210) was obtained from Enzo Life Sciences (Lausen, Switzerland), actin antibody (I-19) and goat anti-rabbit IgG-HRP from Santa Cruz Biotechnology and p-CREB (S133) (87G3) from Cell Signaling Technology (Beverly, MA, USA).

### 4.2. Ethics

Animal experiments were carried out in accordance to FELASA guidelines, the Estonian Law of Animal Protection and were approved by the Authorization Committee of Animal Experiments at Estonian Ministry of Agriculture (22.05.2014, permit no. 33). Animals were housed in the animal facility of the Institute of Molecular and Cell Biology, University of Tartu (ethical permission code KL1202, 10.01.2005). Animals used in the experiments were killed by cervical dislocation.

### 4.3. Animals and Granulosa Cell Isolation

Female C57BL/6J mice used in this study were housed on a 12 h light/12 h dark cycle at stable room temperature. Mice were provided food and water ad libitum. 22–24 day old sexually immature mice were killed immediately (control group) or injected intraperitoneally with 5 IU of PMSG (to induce superovulation), 5 IU of FSH, 5 IU of LH or equivalent volume of vehicle. In combined gonadotropin treatment experiments, mice were injected in total 4 times (every 12 h) with 1.5 IU of FSH and 1.25 IU of LH. The mice were killed immediately or 12–48 h after the first injection and granulosa cells were isolated from ovaries by follicular puncture as previously described [43]. In short, murine ovaries were removed and cleared from connective tissue, washed in cold DMEM/Ham’s F-12 medium, transferred to a tube with preincubation medium (0.5 M sucrose and 10 mM EGTA in DMEM/Ham’s F-12) and incubated at 37 °C until the ovaries precipitated to the bottom of the tube. Subsequently the ovaries were punctured in dissecting medium (0.2% BSA and 10 mM HEPES (pH 7.5) in DMEM/Ham’s F-12 with 26-gauge needles under a light microscope. The cells were collected and pelleted by centrifugation for 5 min at 250× *g*. Isolated cells were harvested immediately for RNA/protein isolation or resuspended in DMEM/Ham’s F-12 medium containing PMSG and/or different reagents and plated on 6-well cell culture dishes coated previously with 1 ug/cm^2^ human fibronectin. Cells were maintained at 37 °C with 5% CO_2_ in an incubator and harvested for mRNA analysis at different timepoints. Alternatively, isolated cells were used for transfection studies (as described below).

### 4.4. Western Blotting

Granulosa cells were lysed with RIPA buffer (50 mM Tris-HCl (pH 7.5), 150 mM NaCl, 0.1% SDS, 0.5% sodium deoxycholate, 1% Triton-X 100) containing 1X protease inhibitor cocktail (Roche, Basel, Switzerland) and resolved on 10% polyacrylamide-SDS gel. Following electrophoresis, proteins were transferred to PVDF membrane (Thermo Scientific, Rockford, IL, USA) and blocked with TBST (20 mM Tris-HCl (pH 7.5), 150 mM NaCl, 0.1% Tween 20) containing 5% non-fat milk. The membrane was incubated overnight at 4 °C with anti-Ahr (SA-210), anti-p-CREB and anti-actin antibodies in TBST milk. ReBlot Plus Strong Antibody Stripping Solution (Merck Serono GmbH, Darmstadt, Germany) was used for stripping (anti-p-CREB) and re-blotting (anti-actin). Goat anti-rabbit secondary IgG-HRP and Immobilon Western chemiluminescent HRP substrate (Millipore, Billerica, MA, USA) were used to detect signals on X-ray films (Agfa, Mortsel, Belgium). The protein bands were quantified using ImageJ software.

### 4.5. Isolation of RNA and mRNA/hnRNA Measurement by RT-qPCR

Blood/Cultured Cell Total RNA Purification Kit (Favorgen, Ping-Tung, Taiwan) was used for RNA extraction followed by DNase I (Thermo Scientific) treatment. Reverse transcription was performed using RevertAid First Strand cDNA Synthesis Kit (Thermo Scientific) followed by quantitative PCR using Maxima SYBR Green qPCR Master Mix (Thermo Scientific) and specific primers (Table 1). PCR cycling conditions were as follows: 95 °C for 10 min, 40 cycles of 95 °C for 10 sec and 60 °C for 1 min. Experiments were performed with LightCycler 480 II Real-Time PCR System (Roche). PCR reactions were performed in two replicates. The results were analyzed with LightCycler 480 software version 1.5 (Roche), using second derivative max values. Target C_p_ values from replicate measurements were averaged and normalized against TATA-binding protein (*Tbp*) reference values. Relative expression level of the target gene (defined as fold change from control C_p_ values) was calculated using the previously described formula [75].

### 4.6. Immunofluorescence

Mouse ovaries were frozen in Tissue Tek O.C.T compound (Sakura Finetek, Alphen aan den Rijn, Netherlands). Seven µm cryosections were cut and mounted on Superfrost Plus adhesion slides (Knittel Glass, Braunschweig, Germany). The tissue sections were fixed with 4% paraformaldehyde, followed by permeabilization with 0.1% Triton X-100. Blocking was performed with 4% normal goat serum (NGS). Ahr antibody (SA-210) in blocking buffer was added to the sections for overnight incubation followed by incubation with secondary antibody Alexa Fluor® 488 goat anti-rabbit IgG. Sections were counterstained with 1 µg/mL DAPI (Sigma-Aldrich, Munich, Germany) and mounted with Fluorsave Reagent (Calbiochem, San Diego, CA, USA). Visualization was performed with Olympus IX81 CellR microscope (Olympus Corporation, Tokyo, Japan).

### 4.7. Plasmids, Transient Transfection and Reporter Gene Assay

The plasmid containing *Ahr* promoter sequence was generated previously [32]. GCs (10^5^ cells per well) were seeded in serum-free medium on a 24-well plate pre-coated with human fibronectin, followed by transfection with 500 ng of pGL3_Ahr-1792 reporter vector or equimolar amount of empty vector (pGL3-basic) after 1h incubation. 50 ng of pSV-b-galactosidase control vector (pSV-b-gal) (Promega, Madison, WI, USA) was used for evaluating transfection efficiency. Transfections were performed using TurboFect in vitro Transfection Reagent (Thermo Scientific). After 12 h, cells were washed with PBS and new medium containing PMSG or vehicle was added. Reporter gene analysis was performed 12 h later with Dual-Light Reporter Assay System (Applied Biosystems, Bedford, MA, USA) according to the manufacturer’s protocol. GloMax 20/20 luminometer (Promega) was used to detect luciferase and b-gal levels. Results are expressed as luciferase/b-gal expression [relative luciferase units (RLU)] and shown as mean fold changes with respect to control (empty pGL3-basic).

### 4.8. Chromatin Accessibility by Real-Time PCR (CHART-PCR)

CHART-PCR was performed as described previously [32]. In short, GCs were allowed to swell on ice for 30 min in buffer A (10 mM HEPES (pH 7.9), 1.5 mM MgCl_2_, 10 mM KCl, 0.25 M sucrose, 0.5 mM DTT, 0.5% BSA, 0.5 mM spermidine and 1× protease inhibitor cocktail), for lysis 0.625% NP-40 was added. Cells were gently suspended through a 26G needle, nuclei were isolated by centrifugation and washed in buffer A. After pelleting, nuclei were resuspended in DNase I buffer (50 mM Tris-HCl (pH 7.5), 0.5% BSA, 0.5 mM spermidine, 1× protease inhibitor cocktail) supplemented with 10 mM MgCl_2_ and 0.5 mM CaCl_2_. Aliquots were made and DNase I was added (0, 10, and 20 units). DNA was digested at 4 °C for 20 min and the reaction was terminated by 25 mM EDTA. Buffer B (100 mM NaCl, 10 mM Tris (pH 8.0), 25 mM EDTA, 0.5% SDS) and 0.1 mg/mL proteinase K (Thermo Scientific) were added and the mixture was incubated at 45 °C overnight. DNA was cleaned with Nucleospin Gel and PCR Clean-up kit (Macherey-Nagel, Düren, Germany). The purified DNA was subjected to qPCR using primers specific to *Ahr*, *Cyp19a1*, and *Pax7* promoters (Table 1). The measured amounts of *Ahr* and *Cyp19a1* were normalized to internal reference, for which a promoter region of developmentally restricted *Pax7* gene was used, previously shown to consistently display reduced chromatin accessibility in various cell lines [76]. Percentage protection from DNase I was calculated as the amount of DNA recovered by qPCR from digested nuclei relative to undigested control. 

### 4.9. Statistical Analysis

Data were obtained from at least three independent experiments. Student t-test was used when comparing mean differences of two experimental groups. For multiple comparisons between treatment groups, one-way ANOVA was used, followed by Tukey’s post-hoc test. The level of statistical significance was established at *p* value of < 0.05. Data are expressed as means ± SD.

## Figures and Tables

**Table 1:** Oligonucleotides used in the study.

Application	Oligonucleotide		Sequence (5′–3′)
qPCR	*Ahr* mRNA	Forward	GGCCAAGAGCTTCTTTGATG
		Reverse	TGCCAGTCTCTGATTTGTGC
	*Ahr* hnRNA E2-I2	Forward	TAGGCTCAGCGTCAGCTACC
		Reverse	GTCACCAACATTTAAAGGACCAC
	*Fshr* mRNA	Forward	GCGGCAAACCTCTGAACTTC
		Reverse	TGATCCCCAGGCTGAGTCAT
	*Cyp19a1* mRNA	Forward	GCCTCCTTCTCCTGATTTGGA
		Reverse	CTGCCATGGGAAATGAGGG
	*Lhcgr* mRNA	Forward	AGTCACAGCTGCACTCTCC
		Reverse	GTGAGAGATAGTCGGGCGAG
	*Tbp* mRNA	Forward	GGCCTCTCAGAAGCATCACT
		Reverse	GCCAAGCCCTGAGCATAA
CHART-PCR	*Ahr* promoter	Forward	TTTTGAGGCTGGAAAACAGGTACT
		Reverse	ACGTGATGACGCAGGACGTA
	*Cyp19a1* promoter	Forward	CCAATCCAGCACCCTTCCAA
		Reverse	GGGAAGAAAGCAGTGGTGGA
	*Pax7* promoter	Forward	GTTATCGGTCCCCTCTCCCT
		Reverse	CTCAACGGCAGGGAAGGG

## Supplementary Materials

The following are available online at https://www.mdpi.com/1422-0067/20/12/3068/s1, Supplementary Figures S1–S5: Supplementary Figure S1. The effect of gonadotropins on gene expression in granulosa cells (GCs) in vivo. Supplementary Figure S2. Immunofluorescence analysis of isotype control for Ahr-specific antibody in ovary following gonadotropin treatment in vivo. Supplementary Figure S3. The effect of forskolin on the expression of follicle maturation marker genes in GCs in vitro. Supplementary Figure S4. The effect of forskolin on *Ahr* expression in GCs in vitro. Supplementary Figure S5. The effect of PMSG on chromatin accessibility at *Pax7* promoter.

## Abbreviations

AHR	aryl hydrocarbon receptor
TCDD	2,3,7,8-tetrachlorodibenzo-p-dioxin
FSH	follicle stimulating hormone
LH	luteinizing hormone
FSK	forskolin
PKA	Protein kinase A
hnRNA	heteronuclear RNA
ActD	actinomycin D
CHART-PCR	chromatin accessibility by real-time PCR

## References

[1] Denison M.S., Nagy S.R. (2003). Activation of the aryl hydrocarbon receptor by structurally diverse exogenous and endogenous chemicals. Annu. Rev. Pharmacol. Toxicol..

[2] Abbott B.D., Schmid J.E., Pitt J.A., Buckalew A.R., Wood C.R., Held G.A., Diliberto J.J. (1999). Adverse reproductive outcomes in the transgenic Ah receptor-deficient mouse. Toxicol. Appl. Pharmacol..

[3] Mimura J., Fujii-Kuriyama Y. (2003). Functional role of AhR in the expression of toxic effects by TCDD. Biochim. Biophys. Acta.

[4] Meyer B.K., Perdew G.H. (1999). Characterization of the AhR−hsp90−XAP2 Core Complex and the Role of the Immunophilin-Related Protein XAP2 in AhR Stabilization. Biochemistry.

[5] Kudo I., Hosaka M., Haga A., Tsuji N., Nagata Y., Okada H., Fukuda K., Kakizaki Y., Okamoto T., Grave E. (2018). The regulation mechanisms of AhR by molecular chaperone complex. J. Biochem..

[6] Zhang L., Savas U., Alexander D.L., Jefcoate C.R. (1998). Characterization of the mouse Cyp1B1 gene. Identification of an enhancer region that directs aryl hydrocarbon receptor-mediated constitutive and induced expression. J. Biol. Chem..

[7] Denison M.S., Fisher J.M., Whitlock J.P. (1989). Protein-DNA interactions at recognition sites for the dioxin-Ah receptor complex. J. Biol. Chem..

[8] Baba T., Mimura J., Gradin K., Kuroiwa A., Watanabe T., Matsuda Y., Inazawa J., Sogawa K., Fujii-Kuriyama Y. (2001). Structure and expression of the Ah receptor repressor gene. J. Biol. Chem..

[9] Hahn M.E. (2002). Aryl hydrocarbon receptors: Diversity and evolution. Chemico-biol. Interact..

[10] Harrill J.A., Hukkanen R.R., Lawson M., Martin G., Gilger B., Soldatow V., Lecluyse E.L., Budinsky R.A., Rowlands J.C., Thomas R.S. (2013). Knockout of the aryl hydrocarbon receptor results in distinct hepatic and renal phenotypes in rats and mice. Toxicol. Appl. Pharmacol..

[11] Gutiérrez-Vázquez C., Quintana F.J. (2018). Regulation of the Immune Response by the Aryl Hydrocarbon Receptor. Immunity.

[12] Barnett K.R., Tomic D., Gupta R.K., Miller K.P., Meachum S., Paulose T., Flaws J.A. (2007). The aryl hydrocarbon receptor affects mouse ovarian follicle growth via mechanisms involving estradiol regulation and responsiveness. Biol. Reprod..

[13] Kolluri S.K., Jin U.H., Safe S. (2017). Role of the aryl hydrocarbon receptor in carcinogenesis and potential as an anti-cancer drug target. Arch. Toxicol..

[14] Kharat I., Saatcioglu F. (1996). Antiestrogenic effects of 2,3,7,8-tetrachlorodibenzo-p-dioxin are mediated by direct transcriptional interference with the liganded estrogen receptor. Cross-talk between aryl hydrocarbon- and estrogen-mediated signaling. J. Biol. Chem..

[15] Son D.S., Ushinohama K., Gao X., Taylor C.C., Roby K.F., Rozman K.K., Terranova P.F. (1999). 2,3,7,8-Tetrachlorodibenzo-p-dioxin (TCDD) blocks ovulation by a direct action on the ovary without alteration of ovarian steroidogenesis: Lack of a direct effect on ovarian granulosa and thecal-interstitial cell steroidogenesis in vitro. Reprod. Toxicol..

[16] Benedict J.C., Lin T.M., Loeffler I.K., Peterson R.E., Flaws J.A. (2000). Physiological role of the aryl hydrocarbon receptor in mouse ovary development. Toxicol. Sci..

[17] Barnett K.R., Tomic D., Gupta R.K., Babus J.K., Roby K.F., Terranova P.F., Flaws J.A. (2007). The aryl hydrocarbon receptor is required for normal gonadotropin responsiveness in the mouse ovary. Toxicol. Appl. Pharmacol..

[18] Benedict J.C., Miller K.P., Lin T.M., Greenfeld C., Babus J.K., Peterson R.E., Flaws J.A. (2003). Aryl hydrocarbon receptor regulates growth, but not atresia, of mouse preantral and antral follicles. Biol. Reprod..

[19] Baba T., Mimura J., Nakamura N., Harada N., Yamamoto M., Morohashi K., Fujii-Kuriyama Y. (2005). Intrinsic function of the aryl hydrocarbon (Dioxin) receptor as a key factor in female reproduction. Mol. Cell. Biol..

[20] Edson M.A., Nagaraja A.K., Matzuk M.M. (2009). The mammalian ovary from genesis to revelation. Endocr. Rev..

[21] Bao B., Garverick H.A. (1998). Expression of steroidogenic enzyme and gonadotropin receptor genes in bovine follicles during ovarian follicular waves: A review. J. Anim. Sci..

[22] Hsueh A.J., Adashi E.Y., Jones P.B., Welsh T.H. (1984). Hormonal regulation of the differentiation of cultured ovarian granulosa cells. Endocr. Rev..

[23] Menon K.M.J., Menon B., Gulappa T. (2018). Regulation of Luteinizing Hormone Receptor mRNA Expression in the Ovary: The Role of miR-122. Vitam. Horm..

[24] Gates A.H., Bozarth J.L. (1978). Ovulation in the PMSG-treated immature mouse: Effect of dose, age, weight, puberty, season and strain (BALB/c, 129 and C129F1 hybrid). Biol. Reprod..

[25] Richards J.S. (1980). Maturation of ovarian follicles: Actions and interactions of pituitary and ovarian hormones on follicular cell differentiation. Physiol. Rev..

[26] Mukherjee A., Park-Sarge O.K., Mayo K.E. (1996). Gonadotropins induce rapid phosphorylation of the 3′,5′-cyclic adenosine monophosphate response element binding protein in ovarian granulosa cells. Endocrinology.

[27] Carlone D.L., Richards J.S. (1997). Functional interactions, phosphorylation, and levels of 3′,5′-cyclic adenosine monophosphate-regulatory element binding protein and steroidogenic factor-1 mediate hormone-regulated and constitutive expression of aromatase in gonadal cells. Mol. Endocrinol..

[28] Puri P., Little-Ihrig L., Chandran U., Law N.C., Hunzicker-Dunn M., Zeleznik A.J. (2016). Protein Kinase A: A Master Kinase of Granulosa Cell Differentiation. Sci. Rep..

[29] Richards J.S., Kersey K.A. (1979). Changes in Theca and Granulosa Cell Function in Antral Follicles Developing during Pregnancy in the Rat: Gonadotropin Receptors, Cyclic AMP and Estradiol-17β. Biol. Reprod..

[30] Chaffin C.L., Stouffer R.L., Duffy D.M. (1999). Gonadotropin and steroid regulation of steroid receptor and aryl hydrocarbon receptor messenger ribonucleic acid in macaque granulosa cells during the periovulatory interval. Endocrinology.

[31] Chaffin C.L., Trewin A.L., Hutz R.J. (2000). Estrous cycle-dependent changes in the expression of aromatic hydrocarbon receptor (AHR) and AHR-nuclear translocator (ARNT) MRNAs in the rat ovary and liver. Chem. Biol. Interact..

[32] Teino I., Matvere A., Kuuse S., Ingerpuu S., Maimets T., Kristjuhan A., Tiido T. (2014). Transcriptional repression of the Ahr gene by LHCGR signaling in preovulatory granulosa cells is controlled by chromatin accessibility. Mol. Cell. Endocrinol..

[33] LaVoie H.A. (2005). Epigenetic control of ovarian function: The emerging role of histone modifications. Mol. Cell Endocrinol..

[34] Garrison P.M., Rogers J.M., Brackney W.R., Denison M.S. (2000). Effects of histone deacetylase inhibitors on the Ah receptor gene promoter. Arch. Biochem. Biophys..

[35] Ko C.I., Wang Q., Fan Y., Xia Y., Puga A. (2014). Pluripotency factors and Polycomb Group proteins repress aryl hydrocarbon receptor expression in murine embryonic stem cells. Stem Cell Res..

[36] Peng X.R., Hsueh A.J., LaPolt P.S., Bjersing L., Ny T. (1991). Localization of luteinizing hormone receptor messenger ribonucleic acid expression in ovarian cell types during follicle development and ovulation. Endocrinology.

[37] LaPolt P.S., Tilly J.L., Aihara T., Nishimori K., Hsueh A.J. (1992). Gonadotropin-induced up- and down-regulation of ovarian follicle-stimulating hormone (FSH) receptor gene expression in immature rats: Effects of pregnant mare’s serum gonadotropin, human chorionic gonadotropin, and recombinant FSH. Endocrinology.

[38] Sites C.K., Patterson K., Jamison C.S., Degen S.J., LaBarbera A.R. (1994). Follicle-stimulating hormone (FSH) increases FSH receptor messenger ribonucleic acid while decreasing FSH binding in cultured porcine granulosa cells. Endocrinology.

[39] Richards J.S. (1994). Hormonal control of gene expression in the ovary. Endocr. Rev..

[40] Richards J.S., Fitzpatrick S.L., Clemens J.W., Morris J.K., Alliston T., Sirois J. (1995). Ovarian cell differentiation: A cascade of multiple hormones, cellular signals, and regulated genes. Rec. Prog. Horm. Res..

[41] Findlay J.K., Drummond A.E. (1999). Regulation of the FSH Receptor in the Ovary. Trend. Endocrinol. Metab..

[42] Chakraborty P., Roy S.K. (2015). Expression of FSH receptor in the hamster ovary during perinatal development. Mol. Cell. Endocrinol..

[43] Teino I., Kuuse S., Ingerpuu S., Maimets T., Tiido T. (2012). The aryl hydrocarbon receptor regulates mouse Fshr promoter activity through an E-box binding site. Biol. Reprod..

[44] McGee E.A., Hsueh A.J. (2000). Initial and cyclic recruitment of ovarian follicles. Endocr. Rev..

[45] Salvador L.M., Park Y., Cottom J., Maizels E.T., Jones J.C., Schillace R.V., Carr D.W., Cheung P., Allis C.D., Jameson J.L. (2001). Follicle stimulating hormone stimulates protein kinase A-mediated histone H3 phosphorylation and acetylation leading to select gene activation in ovarian granulosa cells. J. Biol. Chem..

[46] Gonzalez-Robayna I.J., Alliston T.N., Buse P., Firestone G.L., Richards J.S. (1999). Functional and subcellular changes in the A-kinase-signaling pathway: Relation to aromatase and Sgk expression during the transition of granulosa cells to luteal cells. Mol. Endocrinol..

[47] Ranta T., Knecht M., Darbon J.M., Baukal A.J., Catt K.J. (1984). Induction of granulosa cell differentiation by forskolin: Stimulation of adenosine 3′,5′-monophosphate production, progesterone synthesis, and luteinizing hormone receptor expression. Endocrinology.

[48] Hunzicker-Dunn M., Maizels E.T. (2006). FSH signaling pathways in immature granulosa cells that regulate target gene expression: Branching out from protein kinase A. Cell. Signal..

[49] Minegishi T., Tano M., Kishi H., Kameda T., Miyamoto K. (1997). Follicle-stimulating hormone regulation on its receptor messenger ribonucleic acid levels in cultured rat granulosa cells. Biochim. Biophys. Acta..

[50] Fitzgerald C.T., Nebert D.W., Puga A. (1998). Regulation of mouse Ah receptor (Ahr) gene basal expression by members of the Sp family of transcription factors. DNA Cell Biol..

[51] Garrison P.M., Denison M.S. (2000). Analysis of the murine AhR gene promoter. J. Biochem. Mol. Toxicol..

[52] Zhang J., Watson A.J., Probst M.R., Minehart E., Hankinson O. (1996). Basis for the loss of aryl hydrocarbon receptor gene expression in clones of a mouse hepatoma cell line. Mol. Pharmacol..

[53] Englert N.A., Turesky R.J., Han W., Bessette E.E., Spivack S.D., Caggana M., Spink D.C., Spink B.C. (2012). Genetic and epigenetic regulation of AHR gene expression in MCF-7 breast cancer cells: Role of the proximal promoter GC-rich region. Biochem. Pharmacol..

[54] Lee L., Asada H., Kizuka F., Tamura I., Maekawa R., Taketani T., Sato S., Yamagata Y., Tamura H., Sugino N. (2013). Changes in histone modification and DNA methylation of the StAR and Cyp19a1 promoter regions in granulosa cells undergoing luteinization during ovulation in rats. Endocrinology.

[55] Bussmann U.A., Baranao J.L. (2006). Regulation of aryl hydrocarbon receptor expression in rat granulosa cells. Biol. Reprod..

[56] Combarnous Y., Guillou F., Martinat N., Cahoreau C. (1984). Origin of the FSH + LH double activity of equine chorionic gonadotropin (eCG/PMSG). Ann. Endocrinol..

[57] Palermo R. (2007). Differential actions of FSH and LH during folliculogenesis. Reprod. Biomed. Online..

[58] Ruman J.I., Pollak S., Trousdale R.K., Klein J., Lustbader J.W. (2005). Effects of long-acting recombinant human follicle-stimulating hormone analogs containing N-linked glycosylation on murine folliculogenesis. Fertil. Steril..

[59] Khan H.A., Zhao Y., Wang L., Li Q., Du Y.A., Dan Y., Huo L.J. (2015). Identification of miRNAs during mouse postnatal ovarian development and superovulation. J. Ovar. Res..

[60] Li Y., Fang Y., Liu Y., Yang X. (2015). MicroRNAs in ovarian function and disorders. J. Ovar. Res..

[61] Bahrami A., Miraie-Ashtiani S.R., Sadeghi M., Najafi A. (2017). miRNA-mRNA network involved in folliculogenesis interactome: Systems biology approach. Reproduction.

[62] Carletti M.Z., Christenson L.K. (2009). Rapid effects of LH on gene expression in the mural granulosa cells of mouse periovulatory follicles. Reproduction.

[63] Fiedler S.D., Carletti M.Z., Hong X., Christenson L.K. (2008). Hormonal regulation of MicroRNA expression in periovulatory mouse mural granulosa cells. Biol. Reprod..

[64] Huang T.C., Chang H.Y., Chen C.Y., Wu P.Y., Lee H., Liao Y.F., Hsu W.M., Huang H.C., Juan H.F. (2011). Silencing of MiR-124 induces neuroblastoma SK-N-SH cell differentiation, cell cycle arrest and apoptosis through promoting AHR. FEBS Lett..

[65] Chowdhary V., Teng K.Y., Thakral S., Zhang B., Lin C.H., Wani N., Bruschweiler-Li L., Zhang X., James L., Yang D. (2017). miRNA-122 Protects Mice and Human Hepatocytes from Acetaminophen Toxicity by Regulating Cytochrome P450 Family 1 Subfamily A Member 2 and Family 2 Subfamily E Member 1 Expression. Am. J. Pathol..

[66] Liu C.C., Xia M., Zhang Y.J., Jin P., Zhao L., Zhang J., Li T., Zhou X.M., Tu Y.Y., Kong F. (2018). Micro124-mediated AHR expression regulates the inflammatory response of chronic rhinosinusitis (CRS) with nasal polyps. Biochem. Biophys. Res. Commun..

[67] Harper P.A., Riddick D.S., Okey A.B. (2006). Regulating the regulator: Factors that control levels and activity of the aryl hydrocarbon receptor. Biochem. Pharmacol..

[68] Shi Z., Valdez K.E., Ting A.Y., Franczak A., Gum S.L., Petroff B.K. (2007). Ovarian endocrine disruption underlies premature reproductive senescence following environmentally relevant chronic exposure to the aryl hydrocarbon receptor agonist 2,3,7,8-tetrachlorodibenzo-p-dioxin. Biol. Reprod..

[69] Wigglesworth K., Lee K.B., Emori C., Sugiura K., Eppig J.J. (2015). Transcriptomic diversification of developing cumulus and mural granulosa cells in mouse ovarian follicles. Biol. Reprod..

[70] Maizels E.T., Mukherjee A., Sithanandam G., Peters C.A., Cottom J., Mayo K.E., Hunzicker-Dunn M. (2001). Developmental regulation of mitogen-activated protein kinase-activated kinases-2 and -3 (MAPKAPK-2/-3) in vivo during corpus luteum formation in the rat. Mol. Endocrinol..

[71] Elferink C.J., Reiners J.J. (1996). Quantitative RT-PCR on CYP1A1 heterogeneous nuclear RNA: A surrogate for the in vitro transcription run-on assay. Biotechniques.

[72] Wu C. (1997). Chromatin remodeling and the control of gene expression. J. Biol. Chem..

[73] DeManno D.A., Cottom J.E., Kline M.P., Peters C.A., Maizels E.T., Hunzicker-Dunn M. (1999). Follicle-stimulating hormone promotes histone H3 phosphorylation on serine-10. Mol. Endocrinol..

[74] Collas P., Le Guellec K., Taskén K. (1999). The A-kinase-anchoring protein AKAP95 is a multivalent protein with a key role in chromatin condensation at mitosis. J. Cell Biol..

[75] Livak K.J., Schmittgen T.D. (2001). Analysis of relative gene expression data using real-time quantitative PCR and the 2(-Delta Delta C(T)) method. Methods.

[76] Cruickshank M., Fenwick E., Abraham L.J., Ulgiati D. (2008). Quantitative differences in chromatin accessibility across regulatory regions can be directly compared in distinct cell-types. Biochem. Biophys. Res. Commun..

