# Supplementary material for: FSH/LH-Dependent Upregulation of Ahr in Murine Granulosa Cells Is Controlled by PKA Signaling and Involves Epigenetic Regulation
Source: Int J Mol Sci. 2019 Jun 23;20(12):3068. doi: 10.3390/ijms20123068 (PMC6627912; doi:10.3390/ijms20123068)
Supplement: Supplementary file 1 [file ijms-20-03068-s001.pdf]

Supplementary Figures S1-S5.

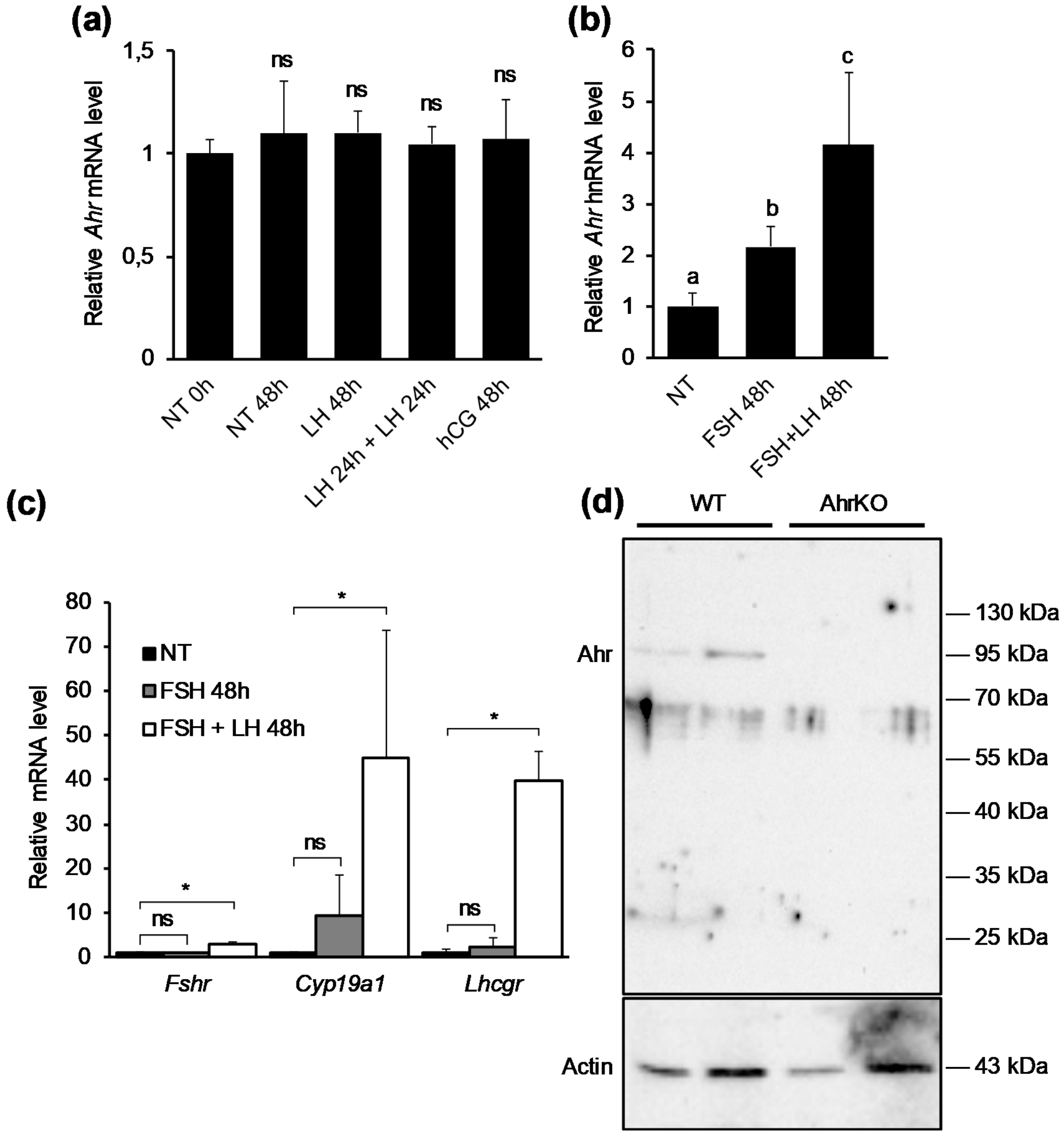

Supplementary Figure S1. The effect of gonadotropins on *gene* expression in granulosa cells (GCs) *in vivo*. (**a**) Mice were injected once (LH 48h, hCG 48h) or twice (LH 24h + LH 24h) with 5 IU of LH, hCG or vehicle (NT) and lysates were collected from GCs isolated before (0 h) or 48 h later. *Ahr* mRNA levels were measured by qPCR. Data are presented as means ± SD from three independent experiments. (**b**) Mice were injected in total 4 times (every 12 h) with FSH (1.5 IU) or FSH (1.5 IU) + LH (1.25 IU) and lysates were collected from GCs isolated before (NT) or 48 h after initial injection. *Ahr* hnRNA levels were measured by qPCR. Data are presented as means ± SD from three independent experiments. (**c**) Mice were injected in total 4 times (every 12 h) with FSH (1.5 IU) or FSH (1.5 IU) + LH (1.25 IU) and lysates were collected from GCs isolated before (NT) or 48 h after initial injection. *Fshr, Cyp19a1 and Lhcgr* mRNA levels were measured by qPCR. Data are presented as means ± SD from three independent experiments. (**d**) Western blot analysis of Ahr antibody using WT and AhrKO mice. ns – non-

significant difference from NT 0h ($p > 0.05$). Bars with different superscripts are significantly different ($p < 0.05$). *$p < 0.05$ vs NT.

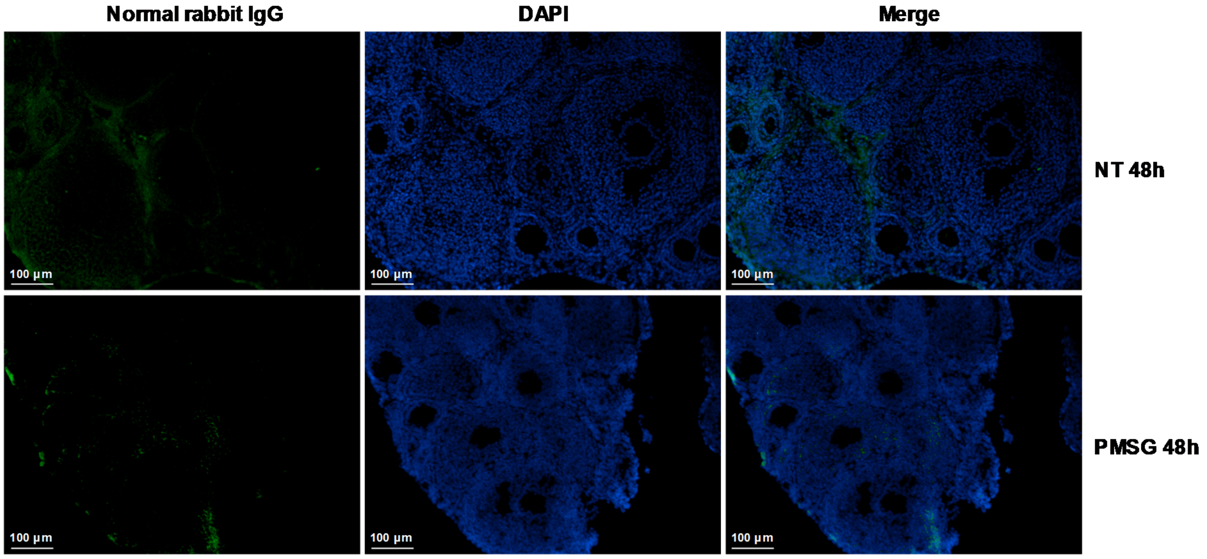

Supplementary Figure S2. Immunofluorescence analysis of isotype control for Ahr-specific antibody in ovary following gonadotropin treatment *in vivo*. Mice were injected with 5 IU of PMSG or vehicle (NT) and ovaries were isolated 48 h later. Representative images of immunofluorescence analysis using Normal rabbit IgG as primary antibody (green). Nuclei were stained with DAPI (blue). Scale bar, 100 µm.

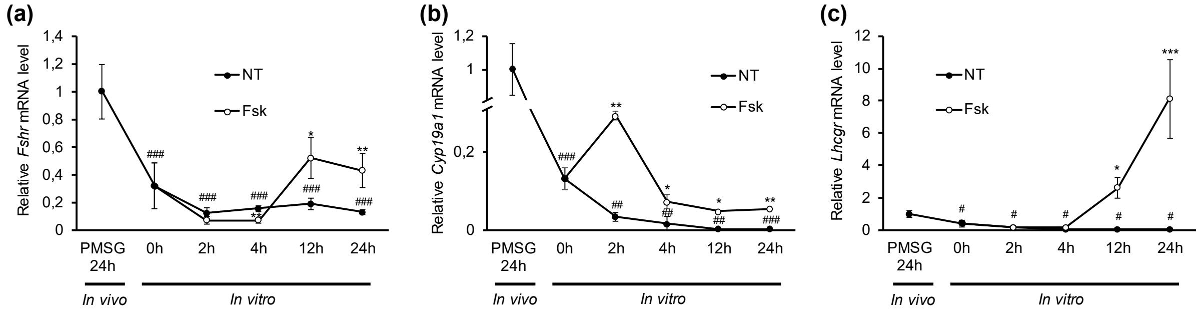

Supplementary Figure S3. The effect of forskolin on the expression of follicle maturation marker genes in GCs *in vitro*. Mice were injected with 5 IU of PMSG and GCs were isolated 24 h later, at which point GCs were subjected to qPCR analysis or cultured *in vitro*. Following attachment (2-3 h later), GCs were treated with vehicle (NT) or Fsk (10 µM) for up to 24 h. *Fshr* (**a**), *Cyp19a1* (**b**) and *Lhcgr* (**c**) levels were measured by qPCR before (0 h) or at different timepoints after Fsk or vehicle was added to medium. Presented values are normalized to *Tbp* expression levels. Data are presented as means ± SD from three independent experiments. *, ** and *** indicate values of $p < 0.05$, $p < 0.01$ and $p < 0.001$ vs NT, respectively. #, ## and ### indicate values of $p < 0.05$, $p < 0.01$ and $p < 0.001$ vs PMSG 24 h *in vivo*, respectively.

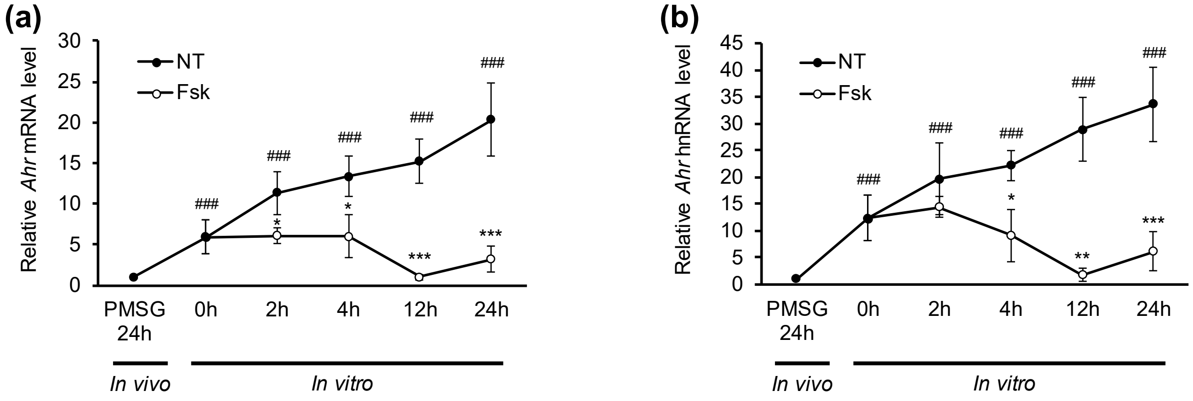

Supplementary Figure S4. The effect of forskolin on *Ahr* expression in GCs *in vitro*. Mice were injected with 5 IU of PMSG and GCs were isolated 24 h later, at which point GCs were subjected to qPCR analysis or cultured *in vitro*. Following attachment (2-3 h later), GCs were treated with vehicle (NT) or Fsk (10 μM) for up to 24 h. *Ahr* mRNA (**a**) and hnRNA (**b**) levels were measured by qPCR before (0 h) or at different timepoints after Fsk or vehicle was added to medium. Data are presented as means ± SD from three independent experiments. *, ** and *** indicate values of $p < 0.05$, $p < 0.01$ and $p < 0.001$ vs NT, respectively. #, ## and ### indicate values of $p < 0.05$, $p < 0.01$ and $p < 0.001$ vs PMSG 24 h *in vivo*, respectively.

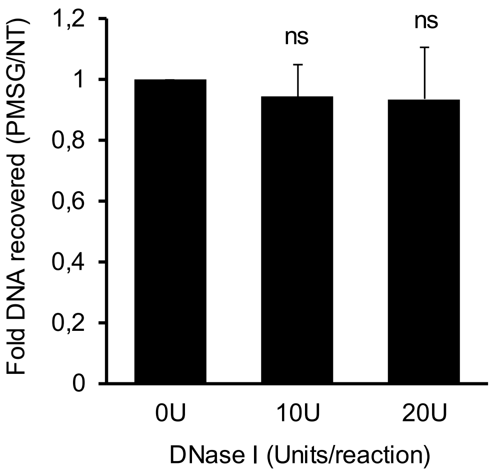

Supplementary Figure S5. The effect of PMSG on chromatin accessibility at *Pax7* promoter. Mice were injected with 5 IU of PMSG or vehicle (NT) and GCs were isolated 48 h later. DNA from nuclei treated with increasing concentrations of DNase I was analyzed by qPCR. The data are presented as ratio of PMSG vs NT of recovered DNA. Data are presented as means ± SD from three independent experiments. ns – non-significant difference from 0 U ($p > 0.05$).
